# Treatment of a Symptomatic Carotid Web: A Case Report and Review of the Literature

**DOI:** 10.7759/cureus.112031

**Published:** 2026-07-04

**Authors:** Mahetab Shehata, Sui Tee, Ashraf Elsharkawy

**Affiliations:** 1 Vascular Surgery, University Hospitals Coventry and Warwickshire NHS Trust, Coventry, GBR

**Keywords:** carotid arterial disease, carotid endarterectomy (cea), carotid web, cerebro-vascular accident (stroke), extracranial fibromuscular dysplasia

## Abstract

A carotid web is identified as an atypical variant of fibromuscular dysplasia. It is characterised by fibrotic, non-atherosclerotic bands found in the carotid artery intima, forming a shelf-like intraluminal web that, in most cases, lies on the posterior wall of the carotid bulb or the proximal internal carotid artery. That shelf formed a site for thrombus accumulation, with a significant risk of further distal embolisation to the cranial circulation, leading to recurrent thromboembolic stroke and transient ischaemic attack. In this case report, a 48-year-old male presented with recurrent transient ischaemic attacks and stroke despite being on the best medical treatment in the form of dual antiplatelets and a high dose of statins. During the most recent event, he presented with left-sided weakness and slurred speech and underwent urgent thrombolysis with full recovery, then was referred to the vascular surgery department. During his workup investigations with carotid duplex and computed tomographic angiography, a carotid web was identified on the posterior wall of the proximal internal carotid artery with 50% stenosis. As per the recent literature consensus and guidelines, due to recurrent symptomatic events despite medical therapy, he was scheduled for carotid endarterectomy with bovine patch repair in the nearest slot.

## Introduction

Carotid web is identified as a variant of fibromuscular dysplasia, characterised by being thrombogenic due to blood stasis in this shelf, leading to thrombus formation and distal trash embolisation. A carotid web is one of the most common causes of stroke and transient ischaemic attacks in young people [1]. A recent systematic review, including 37 articles with 158 patients, demonstrated that carotid webs had a significant prevalence in young women by 68%, as well as in the African race by 70%. Most of the imaging modalities illustrated that a carotid web caused 50% stenosis in 84% of patients, and 56% of them presented with recurrent stroke and transient ischaemic attacks [2]. Although the sensitivity and specificity of the different imaging modalities, including duplex ultrasonography, computed tomography angiography, and high-resolution magnetic resonance angiography, were significantly high, a carotid web might be intractable to detect. Challenges encountered in diagnosing a carotid web were due to a lack of awareness of this pathology with regard to imaging interpretation.

Computed tomography angiography is considered the most valuable imaging modality in carotid web diagnosis, while duplex ultrasound could be informative in providing data with regard to web morphology and identifying haemodynamic changes and peak systolic velocity changes, as well as commenting on thrombus formation. However, it is an operator-dependent modality that requires experience, thereby decreasing its sensitivity and specificity [3].

Despite the significant impact of carotid webs on stroke incidence in young patients with their dramatic consequences, there were no clear evidence-based guidelines regarding the management approach and no available level A evidence at present for the efficiency of single antiplatelets versus dual antiplatelet treatment versus anticoagulation therapy for medical management of carotid webs. Recently published guidelines stated that patients with symptomatic carotid webs had a significant risk of stroke. Recent guidelines also recommended that patients who presented with recurrent strokes or transient ischaemic attacks despite taking the best medical treatment should be offered carotid surgical intervention, either with carotid stenting or carotid endarterectomy [4]. A published meta-analysis by Patel et al. in 2022 concluded that the rate of recurrent stroke and transient ischaemic attack in patients diagnosed with a carotid web and treated medically is 56% compared to a low incidence of 3-6% in patients who underwent carotid endarterectomy or carotid stenting [5].

## Case presentation

**Figure 1:**
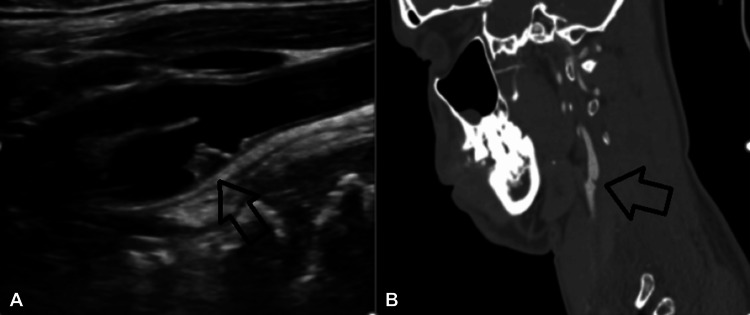
(A) Carotid DUS with an arrow pointing at a carotid web on the proximal ICA. (B) CTA showing healthy, non-calcified arteries, with an arrow pointing at focal narrowing of the proximal ICA DUS: duplex ultrasound; ICA: internal carotid artery; CTA: computed tomography angiography

A 48-year-old African male was admitted to the stroke unit for recurrent ischaemic strokes despite being on the best medical treatment in the form of dual antiplatelet therapy and a high dose of statins. His first event was in July 2025; it presented with left-sided weakness and resolved spontaneously after 20 minutes. The second event occurred in December 2025, presenting with left upper and lower limb weakness, dysarthria, and left facial drop. He was admitted to the stroke unit and was given intravenous recombinant tissue plasminogen activator and physiotherapy rehabilitation, and his symptoms and signs resolved gradually after this treatment. After this event, he was referred to the vascular surgery department. When he was investigated, carotid duplex ultrasound showed a pocket-like structure on the posterior wall of the right proximal internal carotid artery causing 40-50% stenosis, and computed tomography angiography showed proximal right internal carotid artery focal narrowing and a shelf-like structure - a carotid web - causing 50% stenosis (Figure 1) with patent and healthy vessels with no visible calcified plaques in the common carotid artery, external carotid artery and rest of the internal carotid artery bilaterally and patent vertebral arteries on both sides, with a normal anatomical variant where the left internal carotid artery originates from the brachiocephalic artery.

**Figure 2:**
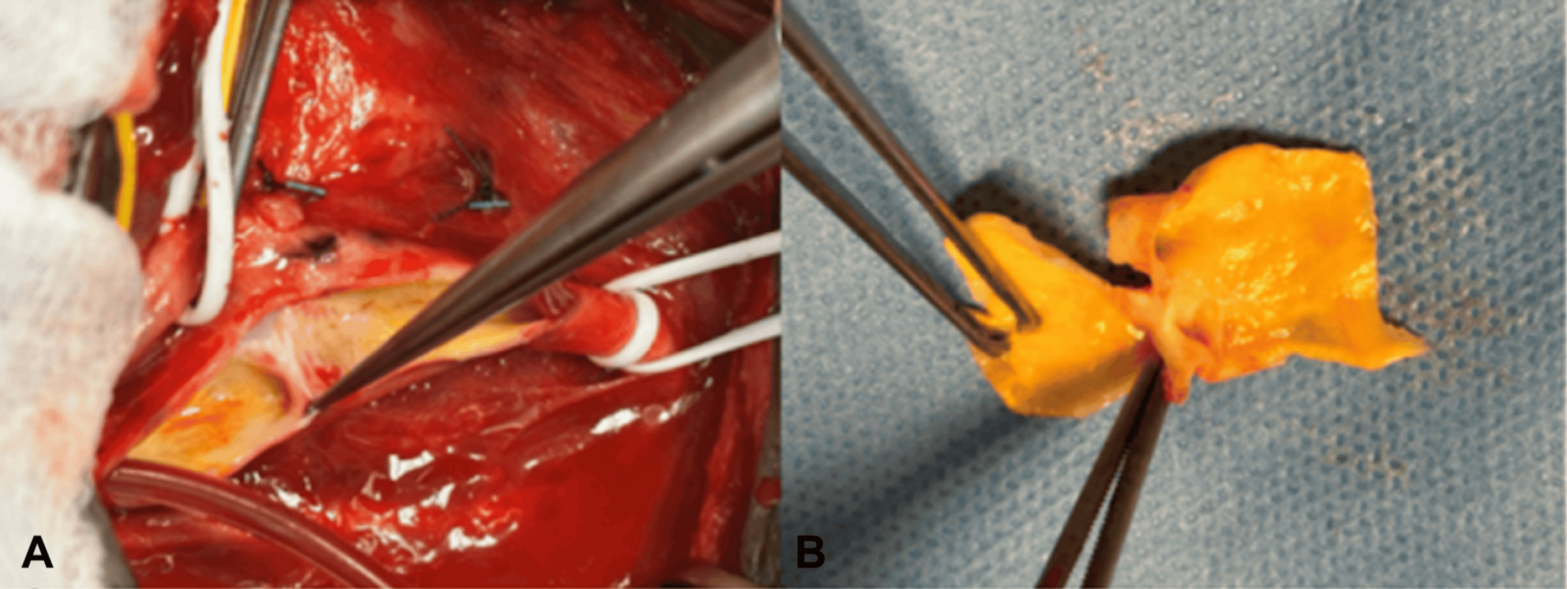
(A) Intraoperative view of the carotid web. (B) Endarterectomised carotid web

**Figure 3:**
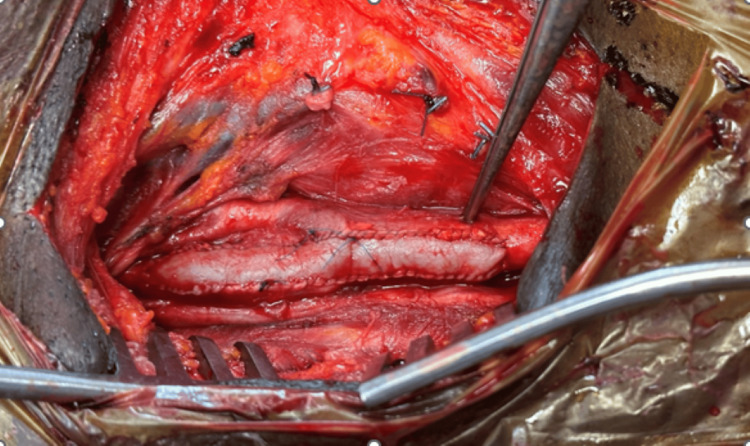
Right carotid endarterectomy with patch repair over the distal CCA and proximal ICA CCA: common carotid artery; ICA: internal carotid artery

On examination, he had no focal neurological deficits or residual weakness, intact vision, a regular pulse without atrial fibrillation, and a right carotid bruit on auscultation. He has a past medical history of hypertension, is on oral antihypertensive medication, and is an ex-smoker who stopped five years ago. He was known to be fit and active for his age and totally independent. He had no previous history of myocardial infarction or angina and had normal kidney function. Due to recurrent neurological events despite being on the best medical treatment, open surgical intervention, such as carotid endarterectomy with bovine patch repair, was offered to the patient, and all possible risks and associated complications were discussed with him, who agreed to proceed. The procedure was performed on the next theatre list, during which the carotid web was successfully endarterectomised with no residual dissection flaps (Figure 2). The endarterectomised right internal carotid artery was repaired with a bovine patch using a shunt (Figure 3).

He had a smooth postoperative recovery, with no surgical complications. He was monitored in the high-dependency unit for postoperative uncontrolled high blood pressure using labetalol and hydralazine intravenous infusion. He was subsequently discharged to the ward after two days on oral antihypertensive medication. He went home with a further follow-up plan as an outpatient, with a total four-day hospital stay.

## Discussion

Because of the unclear prevalence of carotid webs, the MR CLEAN registry in the Netherlands and the CAROWEB registry in France have provided reliable data. The CAROWEB registry included 224 patients; a carotid web was reported during mechanical thrombectomy in 55 of the 85 patients, while the MR CLEAN registry reported a 2.5% prevalence of carotid webs in symptomatic cases and 0.5% in asymptomatic cases in a cohort of 443 patients. The documented symptom recurrence rate was 17% over two years among those managed with medical therapy. These registries also reported that computed tomography angiography predominantly detected carotid webs in young females [6].

Although evidence-based management for the carotid web is not clearly established in the most recent guidelines, recent registries have contributed to the development of the European Society of Vascular Surgeons guidelines. They highlighted that the underdiagnosis of carotid web in initial investigation modalities could be a potential reason for symptom recurrence in most cases. Hence, the guidelines recommended that all symptomatic carotid web cases without any stroke aetiology other than the carotid web could be addressed after an efficient neurovascular workup. Then, carotid intervention, in the form of open surgical endarterectomy or endovascular carotid stenting, might be considered to avoid recurrent transient ischaemic attacks or stroke; this is classified as level C evidence due to the lack of sufficient and consistent evidence-based data. The guidelines also identified carotid web management as an area warranting further research; thus, carotid web patients should be treated on a case-by-case basis, involving the relevant teams: radiologists, stroke physicians, and vascular surgeons [7].

Upon reviewing recent literature on carotid web management, a systematic review and meta-analysis was conducted by the vascular surgery department at Imperial College London in 2025, in which 123 articles were selected and included for data extraction. These 123 articles included two registry reviews, 13 cohort studies, 20 case series, and 73 case reports, spanning 1967 to 2024, and reported 771 patients (registry and cohort studies n=559; case series/case reports n=212). The study found that carotid web prevalence was significantly higher among young females and individuals of Afro-Caribbean descent; it also concluded that the symptom recurrence rate was significantly reduced following carotid intervention, whether by open surgery or stenting [8]. Carotid intervention with definitive management was performed in more than 55.6% of cases; 28 patients (13.2%) underwent carotid stenting, a minimally invasive option used in high-risk patients with dual-layered stents, who achieved good outcomes without significant complications. In comparison, 58 patients (27.3%) underwent open carotid endarterectomy with patch repair. Forty-five of 58 patients' decisions to send intraoperative histology tissue samples were recorded. Results showed subintimal/intimal fibrosis in 32 patients, medial muscular hyperplasia in 12 patients, adventitial fibrosis in two patients, an associated thrombus in 11 cases, arteriosclerosis or plaques in seven cases, myxoid degeneration in eight patients, inflammatory cell infiltration in three patients, and dissection in three patients [9]. Patients who had been treated conservatively with the best medical therapy were associated with a significantly high rate of symptom recurrence, an annual rate of 11.4%. This meta-analysis reported as well that patients with carotid webs presenting with transient ischaemic attacks could worsen to cerebral infarction within three months of medical therapy in two-thirds of the cases, but on the other hand, no symptom recurrence was reported after definitive surgical intervention, either with carotid stenting or carotid endarterectomy with patch repair. The window of time for the definitive surgical intervention also varied and depended on other factors, including symptom recurrence, surgeon preferences, and surgeon case volume. By the end of the literature review, it was concluded that previous studies mainly focused on early diagnosis and the evaluation of management pathways, underscoring the need for further studies with clear evidence-based registries. Worldwide entry would be required to standardise data collection and improve the quality of evidence to guide evidence-based management guidelines [10].

## Conclusions

A carotid web is a challenging pathology that has a significant impact on the young population, as it is recognised as a dramatic cause of recurrent stroke and transient ischaemic attacks. Yet, it remains underdiagnosed on initial neurovascular imaging due to a lack of awareness and evidence-based guidelines. Although there are no formal guidelines for how we should manage and treat this territory of carotid disease, based on the outcomes of recent registries and literature reviews, carotid interventions in the form of open surgical endarterectomy or carotid stenting, depending on patient fitness and general co-morbidities, appear to be a promising and effective definitive treatment for symptomatic cases who present with symptom recurrence despite being on the best medical treatment. Additionally, these interventions are associated with low perioperative risk and decreased annual symptom recurrence rate with improved quality of life.
